# Supplementary material for: Cross-Recognition of Promoters by the Nine SigB Homologues Present in Streptomyces coelicolor A3(2)
Source: Int J Mol Sci. 2021 Jul 22;22(15):7849. doi: 10.3390/ijms22157849 (PMC8346170; doi:10.3390/ijms22157849)
Supplement: Supplementary file 1 [file ijms-22-07849-s001.zip › ijms-1275313-supplementary.pdf]

Supplementary Materials

International Journal of Molecular Sciences; section: Molecular Microbiology; special issue: Bacterial Regulatory Proteins

Cross-recognition of promoters by the nine SigB homologues present in *Streptomyces coelicolor* A3(2)

Beatrica Sevcikova, Bronislava Rezuchova, Vladimira Mazurakova, Dagmar Homerova, Renata Novakova, Lubomira Feckova, Jan Kormanec*

Institute of Molecular Biology, Slovak Academy of Sciences, 845 51 Bratislava, Slovak Republic.

* Correspondence and reprints: jan.kormanec@savba.sk, Tel.: +421 (2) 59307419,
ORCID ID: 0000-0003-0377-6413

Table of contents | Page

Table of contents	Page
Deletion of the *sigB* and *sigH* operons in *S. coelicolor* A3(2)	2
Figure S1: Physical chromosomal DNA maps of *sigB* operon and hybridization analysis.	3
Figure S2: Phenotypic analysis of *S. coelicolor* Δ*sigBop* and Δ*sigB* mutant strains.	4
Figure S3: Physical chromosomal DNA maps of *sigH* operon and hybridization analysis.	5
Figure S4: Phenotypic analysis of *S. coelicolor* Δ*sigHop* and Δ*sigH* mutant strains.	6
Table S1: Oligonucleotides used in this study.	7

Deletion of the sigB and sigH operons in S. coelicolor A3(2)

The entire *rsbB, rsbA, sigB* operon was deleted by the REDIRECT strategy [41] in wild-type *S. coelicolor* M145 strain. The operon was replaced by the apramycin resistance cassette. The strategy resulted in four independent *S. coelicolor sigBop* mutant clones, which were validated by Southern blot hybridization analysis (Supplementary Figure S1). All four validated *S. coelicolor sigBop* mutant strains had a similar phenotype. The *S. coelicolor sigBop* operon mutant was similar to wild-type *S. coelicolor* M145 strain on both rich and minimal media. In addition, we examined the phenotype under osmotic stress conditions (0.5 M NaCl, 1 M sucrose). The *S. coelicolor sigBop* operon mutant was only partially affected (Supplementary Figure S2). Using a similar REDIRECT strategy, we deleted only the *sigB* gene in *S. coelicolor* M145 (Supplementary Figure S1). However, the phenotype of the *sigB* mutant was similar to the *sigBop* operon mutant. Its spores were only slightly paler (Supplementary Figure S2). However, light microscopy revealed that both mutants produced spore chains comparable to the wild-type *S. coelicolor* M145 strain. The growth of both *S. coelicolor sigB* and *sigBop* mutants in liquid NMP medium was also comparable to the parental *S. coelicolor* M145 strain (data not shown).

Another strategy was used to delete the entire *ushY, ushX, sigH* operon in wild-type *S. coelicolor* M145 strain. The operon was replaced with an apramycin resistant cassette flanked by a strong *T4* terminator in a suicide plasmid vector to exclude polar expression. The resulting suicide plasmid pSigHdel3 was used to replace the *ushY, ushX, sigH* operon in wild-type *S. coelicolor* M145 strain by homologous recombination. This strategy resulted in a single mutant *S. coelicolor sigHop* strain, which was validated by Southern blot hybridization analysis (Supplementary Figure S3). The *S. coelicolor sigHop* mutant was phenotypically similar to wild-type *S. coelicolor* M145 strain on rich and minimal solid media. In addition, we examined the phenotype under osmotic stress conditions, and the *S. coelicolor sigHop* operon mutant was also similar to wild-type *S. coelicolor* M145 strain (Supplementary Figure S4). The growth of the *S. coelicolor sigHop* mutant in liquid NMP medium was comparable to the parental *S. coelicolor* M145 strain. In addition, light microscopy revealed that the mutant produced spore chains comparable to the wild-type *S. coelicolor* M145 strain (data not shown).

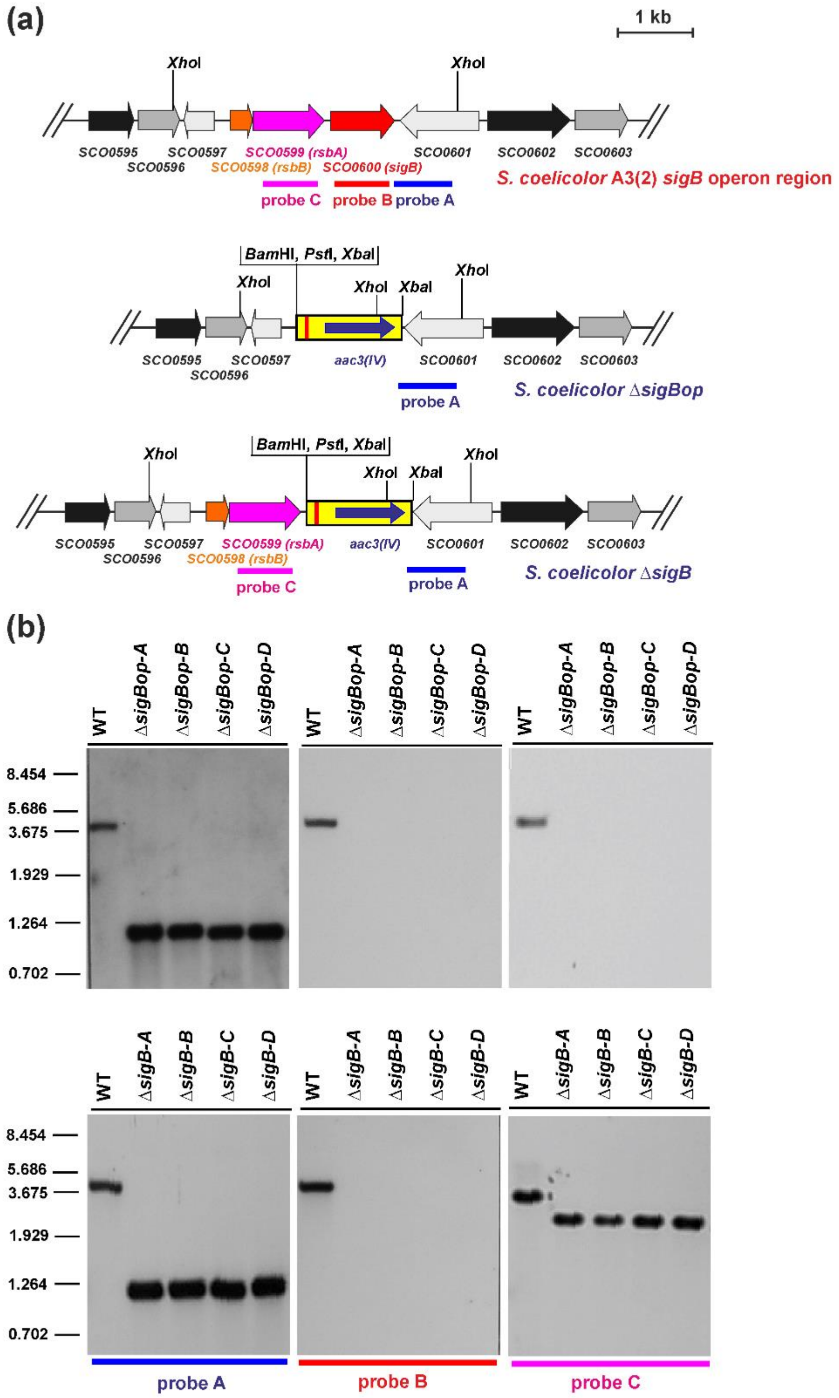

Figure S1. (**a**) Physical chromosomal DNA maps comprising the wild-type *sigB* operon region of *S. coelicolor* M145 and the disrupted alleles of the *sigB* operon and the *sigB* gene in *S. coelicolor* Δ*sigBop* and *S. coelicolor* Δ*sigB*, respectively. The bars below the maps represent the probes used for Southern hybridization analysis. Relevant restriction sites are indicated. (**b**) Southern blot hybridization analysis of chromosomal DNA from four independently obtained *S. coelicolor* Δ*sigBop* and *S. coelicolor* Δ*sigB* clones and wild-type *S. coelicolor* M145 (WT) as reference. 1 μg of DNA from the corresponding strain was digested with the restriction endonuclease *Xho*I, separated by electrophoresis in a 0.8% (w/v) agarose gel, and transferred to a Hybond N membrane (Roche) as described in [49]. Hybridization was performed according to the standard DIG protocol (Roche) using DIG-labelled probes. Lambda DNA-*Bst*EII digest was used as a size standard.

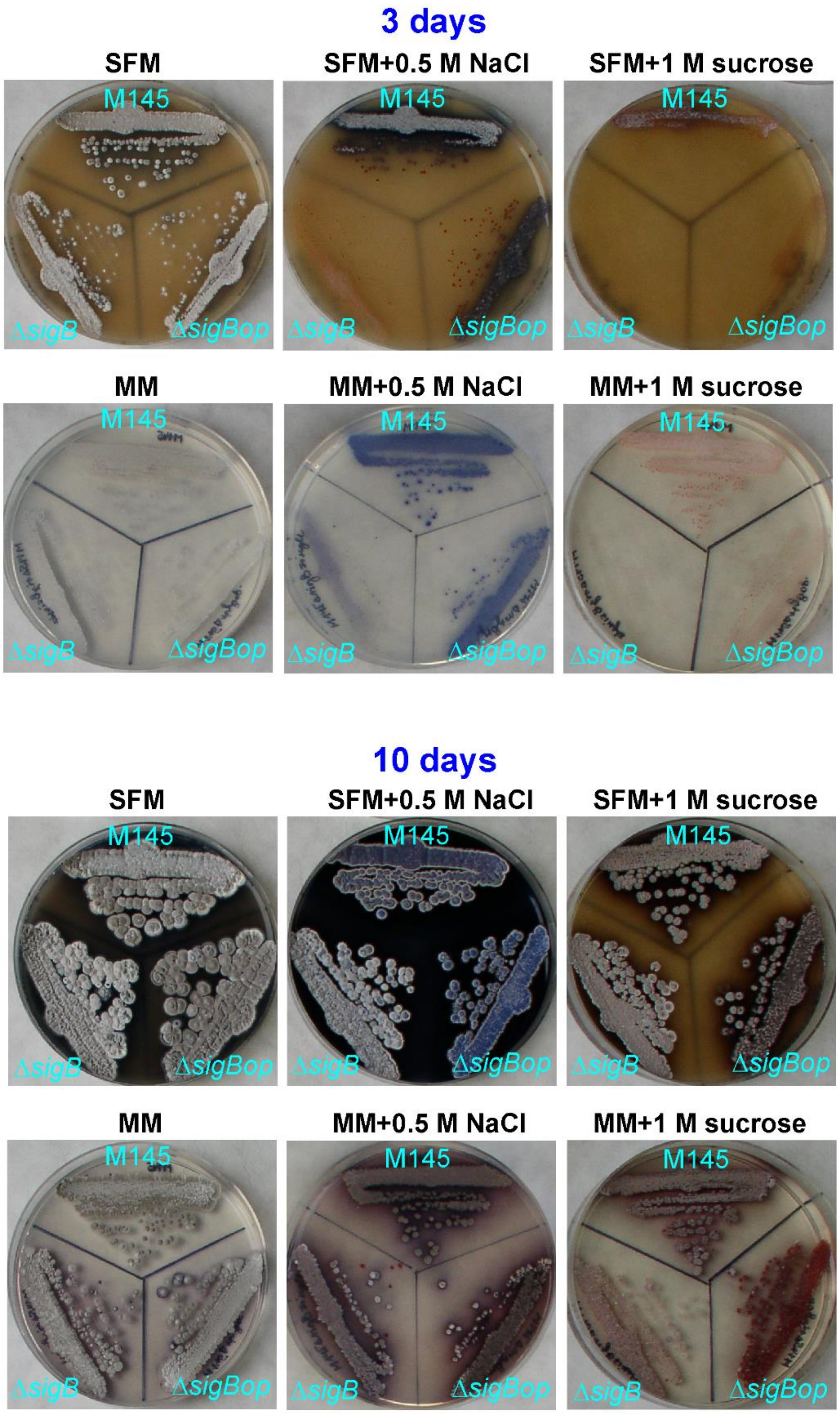

Figure S2. Phenotypic analysis of *S. coelicolor* Δ*sigBop* and *S. coelicolor* Δ*sigB* mutant strains. Spores of the wild-type *S. coelicolor* M145, *S. coelicolor* Δ*sigBop* and *S. coelicolor* Δ*sigB* mutants were spread on the solid rich SFM medium, minimal MM medium containing 0.5% mannitol, both media with 0.5 M NaCl or 1 M sucrose and grown for 3 and 10 days at 28 °C.

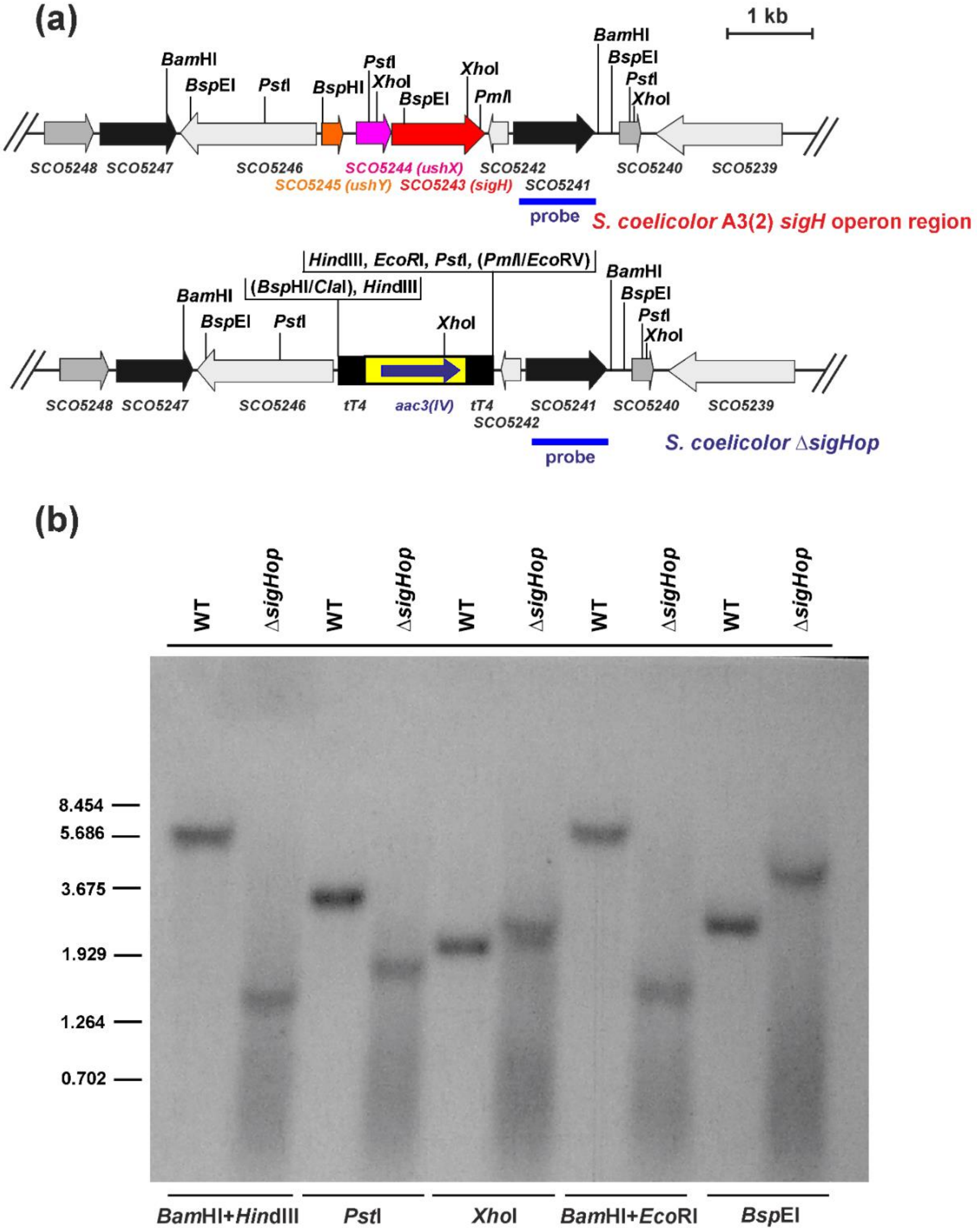

Figure S3. (**a**) Physical chromosomal DNA maps comprising the wild-type *sigH* operon region of *S. coelicolor* M145 and the disrupted allele of the *sigH* operon in *S. coelicolor* Δ*sigHop.* The bar below the maps represents the probe used for Southern hybridization analysis. Relevant restriction sites are indicated. (**b**) Southern blot hybridization analysis of chromosomal DNA from *S. coelicolor* Δ*sigHop* and wild-type *S. coelicolor* M145 (WT) as reference. 1 μg of DNA from the corresponding strain was digested with the indicated restriction enzymes, separated by electrophoresis in a 0.8% (w/v) agarose gel, and transferred to a Hybond N membrane (Roche) as described in [49]. Hybridization was performed according to the standard DIG protocol (Roche) using DIG-labelled probe. Lambda DNA-*Bst*EII digest was used as a size standard.

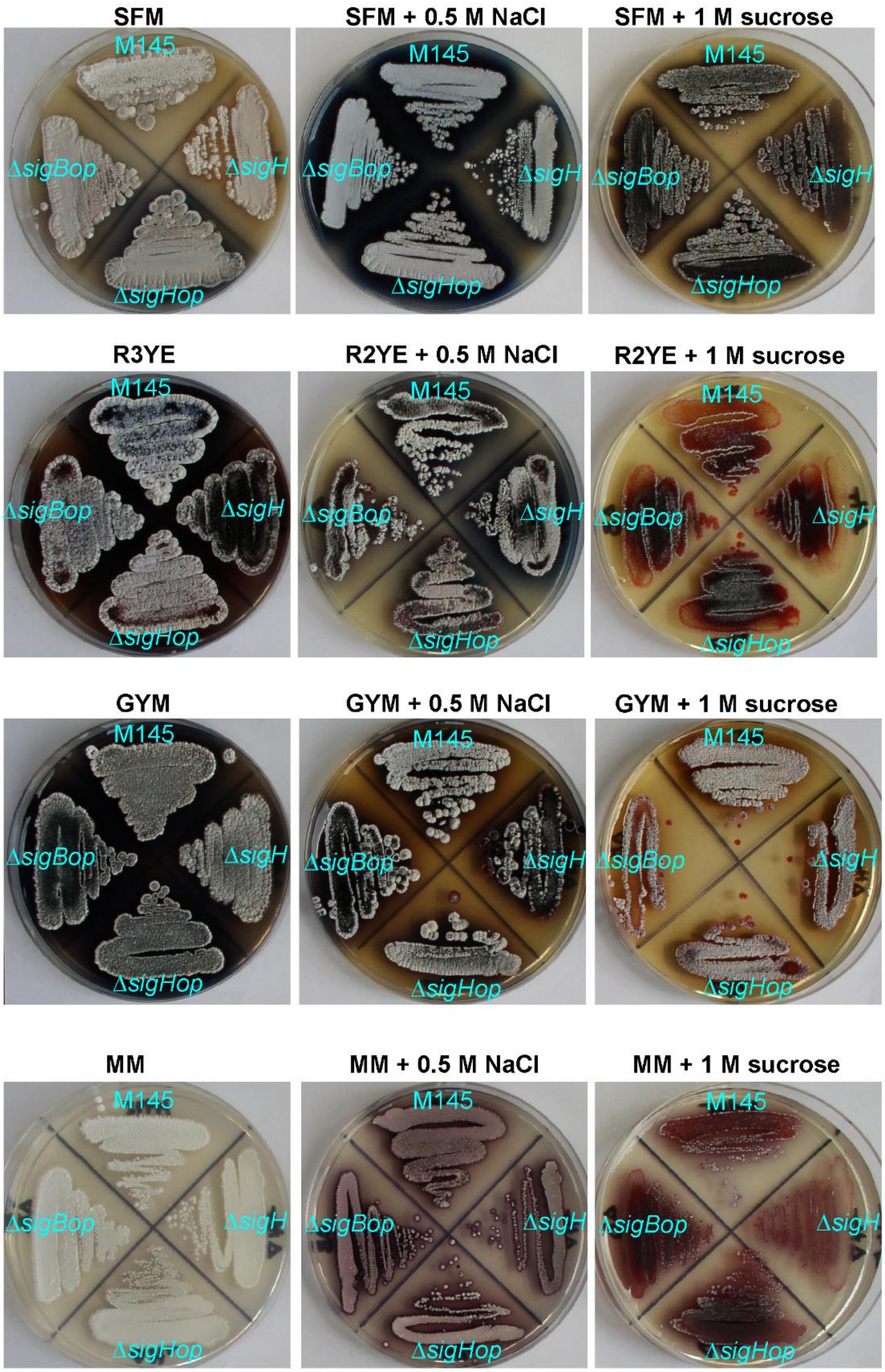

Figure S4. Phenotypic analysis of *S. coelicolor* Δ*sigHop* mutant strain. Spores of the wild-type *S. coelicolor* M145, *S. coelicolor* Δ*sigHop*, *S. coelicolor* Δ*sigBop*, *S. coelicolor* Δ*sigH* [27] prepared in M145 genetic background were spread on the solid rich SFM, R2YE and GYM media and minimal MM medium containing 0.5% mannitol and grown for 10 days at 28 °C. In addition, the same amount of spores was spread on similar media containing 0.5 M NaCl or 1 M sucrose.

Table S1. Oligonucleotides used in this study. Cloning sites are underlined.

Oligonucleotide	Sequence (5′ − 3′)
SigBNde	GGGGCATATGCCGGAGATCGCGGACCCGTCC
SigBHind	GGGAAGCTTCAGGTGGTGCTGAGCATGCCTTCC
SigF2Nde	GGGGCATATGAGCCGGGGCGCCGACACCCGGGC
SigF2Hind	GGGAAGCTTATGCGTCGATCCGGTTCGCGG
SigH2Nde	GGGGCATATGCGGGACGAAGAGCGCGGTACACG
SigH2Hind	GGGAAGCTTACTCCTCGACGAGCAGCTTCTCC
SigINde	GGGGCATATGTCACCCCGGCTCGACGGATCG
SigIHind	GGGAAGCTTATCACTCCTCGACCGTGAGCCCC
SigKNde	GGGGCATATGCCGATCCACGCCAGCGTGAAGC
SigKHind	GGGAAGCTTCACGCCTGCGGACCCGGATCCC
SigLNde	GGGGCATATGCAGACCGCCGTGGTCCGTCCGC
SigLHind	GGGAAGCTTCAGGCGCAGCCGAGTTCGCCCAGC
SigMNde	GGGGCATATGCTCATAGAAACGCCCACCATCC
SigMHind	GGGAAGCTTCACCTGCTGTGTCCCCTGTGATCC
SigNNde	GGGGCATATGTCCGCAGAACAGGGCAGCTCG
SigNHind	GGGAAGCTTCAGTCGGAGATGAGACCCTCGC
−47	CGCCAGGGTTTTCCCAGTCACGAC
Mut80	GGGTTCCGCGCACATTTCCCCG
SCO0566r	GGTCCTGCTCCGCCTCCCGTACC
SCO2512r	AGTGAGGGCAGAACAATTCCTGC
SCO1089r	CCACCACCCTGCTTGCCGGAGG
rrnEr	CCACGAGAGCGGAACAGCCG
SCO0775r	GGTGTCCATCAGCGTCGTGCCC
SCO0279r	GCGGCACCACCTGGTCGAAAAGG
SCO5998r	CGATCCGGGCGATCATCTCAAGG
SCO3557r	GAATGCCGCAGAGCAGTCCGGTCG
SCO7446r	CCAGAAACCGCCGGGCCAGTTC
SCO6509r	GCCACAGGACCAGAACCGCC
SCO1566r	CGAGCACTGCCTTGATGAACAC
SCO2315r	GGACGAACCGCACCAGGTCG
SCO6014r	CGCCGACAGGGATTTCTTGAG
SCO5749r	CCTCCAGCGCAAGCAGATTCTCC
SigBopDir	GCCGGCGTATGCCCGGCGAAGTTCCGAAGGGGA<br>TCGAGTATTCCGGGGATCCGTCGACC
SigBopRev	GAAGAAGACGACGACCGCGGCCTGAGACCCGAG<br>CCCCGCTGTAGGCTGGAGCTGCTTC
SigBDir	GTGACAACGACGACCGATCCACCGGGAGGGCAC<br>ATCATGATTCCGGGGATCCGTCGACC
